# Long Non-coding RNA *ANRIL* in the Nucleus Associates With Periostin Expression in Breast Cancer

**DOI:** 10.3389/fonc.2019.00885

**Published:** 2019-09-11

**Authors:** Paulomi M. Mehta-Mujoo, Heather E. Cunliffe, Noelyn A. Hung, Tania L. Slatter

**Affiliations:** ^1^Department of Pathology, Dunedin School of Medicine, University of Otago, Dunedin, New Zealand; ^2^Maurice Wilkins Centre for Molecular Biodiscovery, Auckland, New Zealand

**Keywords:** LncRNA, *ANRIL*, breast cancer, periostin, CCL2, IL-6, RNAscope

## Abstract

The long non-coding RNA (LncRNA) antisense RNA in the INK4 locus (*ANRIL*) is overexpressed in several cancers including breast cancer. To better understand the role of *ANRIL* in breast cancer this study investigated where *ANRIL* was expressed in breast tumors using *in situ* hybridization by RNAscope. Additional RNAscope assays for *IL6, CCL2*, and *POSTN* were used to establish whether *ANRIL* correlated with increased tumor promoting cytokines. Breast tumors with *ANRIL* over expressed from real-time quantitative (RT-q) PCR assays were selected for analysis using RNAscope. All tumors showed *ANRIL* expression in malignant cells, but amongst tumors *ANRIL* showed different subcellular locations with 56% of tumors with *ANRIL* only in the nucleus, 16% with *ANRIL* only in the cytoplasm and 28% with *ANRIL* in both the nucleus and cytoplasm. Cases with nuclear *ANRIL* were positively correlated with *POSTN* expression in malignant cells (ρ = 0.57, *P* = 0.0086), and no correlation was found between *ANRIL* and *IL6* or *CCL2*. Reduced *POSTN* was also found using siRNA to *ANRIL* in MDA-MB-231 and MCF7 breast cancer cells. These data indicate that *ANRIL* is expressed in malignant breast cells, and suggest its subcellular location may indicate its function in cancer progression.

## Introduction

Given that the progression of breast cancer is associated with poor prognosis, a better understanding of breast cancer progression will better aid targeted patient management and treatment (1). Long non-coding RNAs (LncRNAs) are generally defined as RNA molecules longer than 200 nucleotides with no coding ability (2). They have been proposed to function in multiple cancer pathways including promoting proliferation, migration, invasion, inflammation, and metastasis (2, 3). One such regulatory LncRNA, *ANRIL*, spans over 126 kbp of genomic sequence and three tumor suppressor genes encoded at 9p21 (4–6). In inflammatory conditions including cardiovascular disease and ulcerative colitis *ANRIL* expression correlates with increased expression of three pro-inflammatory cytokines *IL6, CCL2*, and *POSTN* (4, 7–10).

*ANRIL* is also over expressed in many cancers including breast (11–16), prostate (5), colorectal (17), gastric (18), and brain (19). Amongst breast cancers multiple studies have found elevated *ANRIL* in triple negative tumors (12, 14–16). Amongst this subtype, *ANRIL* was elevated in 25–40% of cases, associated with a poorer prognosis, and was included as part of a three non-coding RNA signature proposed for distinguishing triple negative breast cancer from other cancer types (15). Overexpression of *ANRIL* does occur in some estrogen receptor (ER) and progesterone receptor (PR) positive breast cancers. These include tumors from women that carried the minor allele of the rs11515 polymorphism in the 3′ UTR of *cyclin dependent kinase inhibitor 2A* (*CDKN2A*) that encodes both the p16^INK4a^ and p14^*ARF*^ tumor suppressors (13). In addition to increased *ANRIL*, breast tumors from those with the rs11515 minor allele had reduced expression of *p16*^*INK*4*a*^ offering one explanation of how *ANRIL* could be contributing to breast cancer progression. A role for *ANRIL* in promoting breast cancer proliferation was also found when *ANRIL* was knocked down in breast cancer cell lines and tumor xenografts (16).

Despite *ANRIL* being well-studied as a biomarker in breast cancer, it is uncertain if *ANRIL* is expressed within tumors or other cells that are part of the breast tumor microenvironment. The subcellular localization of *ANRIL* and whether it correlates with *IL6, CCL2*, and *POSTN* expression, found in other conditions, in breast cancer is also unknown (4, 7–10). To investigate this, we used RNAscope to examine the cell type and the subcellular location containing *ANRIL* in breast tumors. Whether *ANRIL* expression correlates with *IL6, CCL2*, and *POSTN* in breast tumors was also investigated to provide evidence of how *ANRIL* may contribute to inflammation in breast cancer (20–22).

## Materials and Methods

### Study Cohort

The study cohort consisted of 60 women diagnosed with primary breast cancer who underwent surgery at Dunedin hospital, New Zealand. Clinicopathological data for the cohort is provided in Table 1. Blocks of formalin fixed paraffin embedded material (FFPE) from breast tumor and separate blocks of normal tissue adjacent to tumor tissue were available. Ethical approval was obtained from the Lower South Regional Ethics Committee, Ministry of Health, New Zealand (Ethics Committee Approval LRS/10/09/035) and all participating women gave written and informed consent for inclusion in the study. Standard procedures including culturally appropriate tissue handling and disposal protocols were followed. In addition to the breast cohort, commercially available RNA (Takara Bio, Shiga, Japan) supplied on dry ice and stored at −80°C before use from a variety of tumors and normal tissues were also quantified for *ANRIL* expression. Breast cancers were separated into molecular subtypes (Luminal A, Luminal B, Her 2 positive and triple negative breast cancer) using immunohistochemical surrogates for molecular classifications (23). The luminal A group were classified as ER and PR (≥20%) positive, Her 2 negative and low for Ki67 (<14%); Luminal B were classified as ER and PR (≥20%) positive and high for Ki67 (≥14%), or ER positive with low PR positive cells (<20%); Her 2 positive were all Her 2 positive tumors; and the triple negative breast cancer were classified as all tumors that were negative for ER, PR, and Her 2.

**Table 1:** Clinicopathological data for the breast cancer cohort used in this study.

Age at surgery years (25–75th percentile)	60 (54.25–69.75)
**Ethnicity**	
Maori	2 (3%)
European	54 (90%)
Other	4 (7%)
Lymph/vascular invasion present	18 (30%)
**Metastases to lymph nodes**	
None	28 (47%)
1 node	15 (25%)
≥ 2 nodes	17 (28%)
**Tumor subtype**	
Luminal A	23 (38%)
Luminal B	19 (32%)
Triple negative	7 (12%)
Her 2 positive tumors	11 (18%)

### RNA Extraction

Sections (10 sections at 10 μm) from FFPE breast tumor and normal tissue adjacent to the tumor tissue blocks were subjected to RNA extraction using the RecoverAll™ Total Nucleic Acid Isolation Kit (ThermoFisher Scientific, Waltham, MA, USA) according to the manufacturer's instructions. Eluted RNA was subject to further purification using the RNA Clean-Up and Concentration Kit™ (Norgen Biotek, Thorold, ON, Canada) and incubated at 70°C for 20 min immediately prior to cDNA synthesis (24). High Capacity RNA-to-cDNA Kit (ThermoFisher Scientific, Waltham, MA, USA) was used to convert total RNA to cDNA according to the manufacturer's protocol.

### Quantification of *ANRIL* Using TaqMan Assays

Quantification of *ANRIL* (assay ID Hs01390879_m1) was performed using the Applied Biosystems TaqMan Non-Coding RNA Assay (ThermoFisher Scientific, Waltham, MA, USA) with hypoxanthine phosphoribosyltransferase (HPRT) and glyceraldehyde-3-phosphate dehydrogenase (GAPDH) assays (IDs Hs99999909_m1 and Hs03929097_g, respectively) included as reference genes. Gene expression was quantified in triplicate from single cDNA preparations using the LightCycler 480 (LC480) qPCR machine (Roche, Basel, Switzerland). Relative gene expression was calculated using double delta Ct (ΔΔCT) method and qbase^+^ software (25).

### RNAscope *in situ* Hybridization

RNAscope 2.5 HD Assay-BROWN manual kit™ (Advanced Cell Diagnostics, Newark, CA, USA) was used to measure *ANRIL* (Hs-CDKN2B-AS1), *IL-6* (Hs-IL6)*, CCL2* (Hs-CCL2), and *POSTN* (Hs*-*POSTN) on FFPE tissue sections. Positive (*Ubiquitin C, UBC)* and negative control (the bacterial gene *DapB)* probes were performed on serial tissue sections. Tissue blocks were sectioned at 4 μm thickness and mounted onto Leica BOND™ Plus Slides (Leica Biosystems, Wetzlar, Germany). The assays were performed using the manufacturer's protocol. Stained slides were scanned into the Aperio Scanscope CS digital pathology system slide scanner under 400x magnification (Leica Biosystems, Wetzlar, Germany). The number of dots per cell and the number of positive cells were calculated to provide a score to evaluate the amount of staining present. Expression of *ANRIL* was scored 0–3 as outlined below. A score of 0 (no staining or <1 dot in a total of ten cells), 1 (1–3 dots per cell with <20% of cells per high powered field (hpf, at x400 magnification) positive), 2 (4–9 dots per cell in at least 20% of cells positive or >20% of cells per hpf with at least 1 dot per cell), 3 (>10 dots per cell and at least 20% of cells positive per hpf or >40% of cells with at least 1 dot per cell). For the correlation between *ANRIL* and *POSTN* the average amount of dots per 200 malignant cells were calculated from the average of three fields with 200 cells per field counted. Small clusters of staining were considered to contain ten dots and large clusters 20 dots.

RNAscope was also performed on MCF7 cells treated with siRNA toward *ANRIL* or a control siRNA. Cells from 6 well-plates were combined and made into a cell clot using HistoGel^TM^ specimen processing gel (Thermo Fisher Scientific, Massachusetts, USA). Cell clots were fixed in 10% neutral buffered formaldehyde overnight, and processed into paraffin wax. Cell clot sections (5 μm) were subjected to RNAscope for *ANRIL, POSTN, FBXO18, TERC, PPIB, UBC*, and *DapB* according to the manufacturer's instructions for cells. Cells were imaged using a (Zeiss LSM510; Carl Zeiss, Thornwood, NY, USA), and images were analyzed by Zeiss LSM Image Examiner software version 3.2.0.115 (Carl Zeiss), or imaged using the Aperio Scanscope CS digital pathology system.

### Cell Culture

MCF7 and MDA-MB-231 cell lines (American Type Culture Collection, ATCC) were cultured in Dulbecco's Modified Eagle Medium (DMEM) from Life Technologies (Gibco BRL, Grand Island, NY, USA) and supplemented with 10% fetal bovine serum (FBS) in a 37°C humidified incubator at 5% CO_2_.

### siRNA

Cells were transfected with Lincode Human *CDKN2B-AS1* (R-188105-00-0005) siRNA; Dharmacon, Chicago, IL, USA) using Lipofectamine 2000 (Invitrogen, Burlington, ON, Canada). ON-Target plus Non-targeting Pool (D-001810-10-05) was used as a negative control as suggested by the manufacturer. siRNAs were transfected at a final concentration of 5 or 10 nM using Lipofectamine RNAiMax (Invitrogen). The siRNA and RNAiMax were combined in additional culture media and the resulting lipoplexes were added to the wells with a total of 1 × 10^4^ cells/2 mL for overnight transfection. After 24 h the cultured media was replaced with fresh media and finally harvested at 48 h in total. Each experiment was performed at least in triplicate. *ANRIL* knockdown was verified by real-time RT-PCR using *GAPDH* and *HPRT* as reference genes and tested for *ANRIL* and *POSTN* expression as detailed elsewhere.

### Cytotoxicity Assay

Cells were seeded at a concentration of 1 × 10^5^ cells/mL into a 96-well plate and assayed using the Vybrant MTT Cell Proliferation Assay kit (ThermoFisher Scientific, Waltham, MA, USA) following the manufacturer's protocol for adherent cells.

### Statistical Analysis

Student's *t* test (two tailed) was used to compare two groups, one- way ANOVA for multiple comparisons was used to compare more than two groups, and Pairwise Spearman correlation was performed between *ANRIL* and *POSTN* expression using GraphPad Prism7 software (California, USA). A *P* value < 0.05 was considered statistically significant.

## Results

### *ANRIL* Is Expressed Within Malignant Cells in Different Subcellular Localizations in Breast Tumors

**Figure 1:**
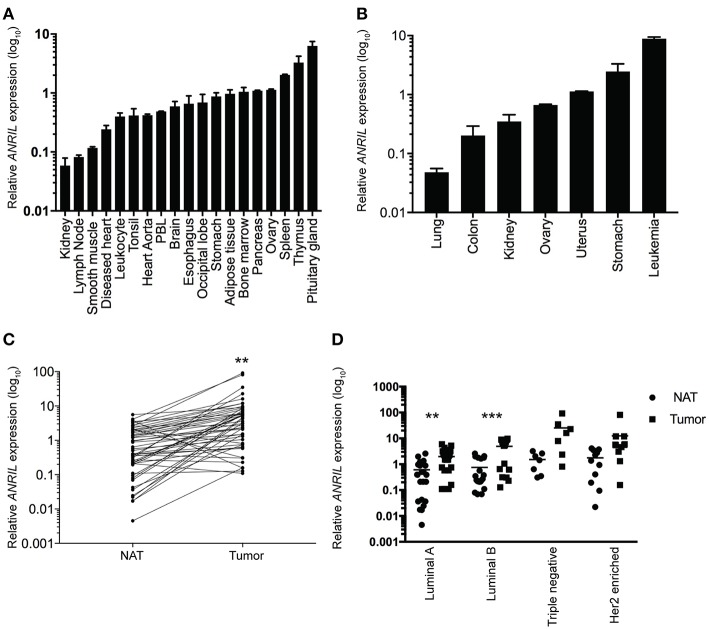
Relative *ANRIL* expression levels in various normal and cancer tissues including primary breast cancer. **(A)** RT-qPCR results from normal commercially available tissues showed a range of *ANRIL* expression levels suggesting *ANRIL* could be expressed as part of the tumor micro environment. **(B)** RT-qPCR results from cancerous commercially available tissues showed a range of *ANRIL* expression levels. Data represented as mean ± SEM for four replicates of each sample. **(C)** Line plot showing higher *ANRIL* levels in breast cancer tissue compared to the matched normal tissue adjacent to the tumor (NAT) from 60 breast cancer patients measured from RT-qPCR assays. *ANRIL* in tumors was significantly higher than NAT (^**^P < 0.01). **(D)** Profile of *ANRIL* expression amongst different tumor subtypes showed in Luminal A and Luminal B tumors *ANRIL* was increased in tumors compared to NAT (^***^P < 0.001). Data represented as mean ± SD for three replicates of each sample. All RT-qPCR normalized to *GAPDH* and *HPRT*.

To determine where *ANRIL* was located in breast tumors we first determined the expression of *ANRIL* in 60 breast tumors, and other tissues from a commercially available source using RT-qPCR. Expression of *ANRIL* was found in all tissues in varying amounts being lowest in lung cancer, kidney, lymph node, and smooth muscle and highest in spleen, thymus, pituitary gland, stomach cancer and leukemia (Figures 1A,B). The presence of *ANRIL* mRNA in a wide variety of tissues including hemopoietic tissues suggested *ANRIL* could be expressed in both tumor cells and the tumor microenvironment. In breast tumors *ANRIL* was increased in RNA extracted from tumor tissue compared to the patient-matched normal tissue adjacent to the tumor *ANRIL* (Figure 1C, *P* = 0.0037). An analysis of *ANRIL* based on breast tumor subtypes compared to the normal associated tissues showed *ANRIL* was increased in Luminal A (Figure 1D, *P* = 0.0017) and Luminal B (Figure 1D, *P* = 0.0008) tumors compared to the normal associated tissue. Although the highest average expression of *ANRIL* was found in triple negative tumors, there was no significant difference in *ANRIL* when compared to the normal associated tissue. A comparison of *ANRIL* amongst tumor tissues showed that *ANRIL* was increased in triple negative tumors compared to Luminal A (*P* = 0.011) and B (*P* = 0.027) with no significance found compared to Her 2 positive tumors (*P* = 0.35).

**Figure 2:**
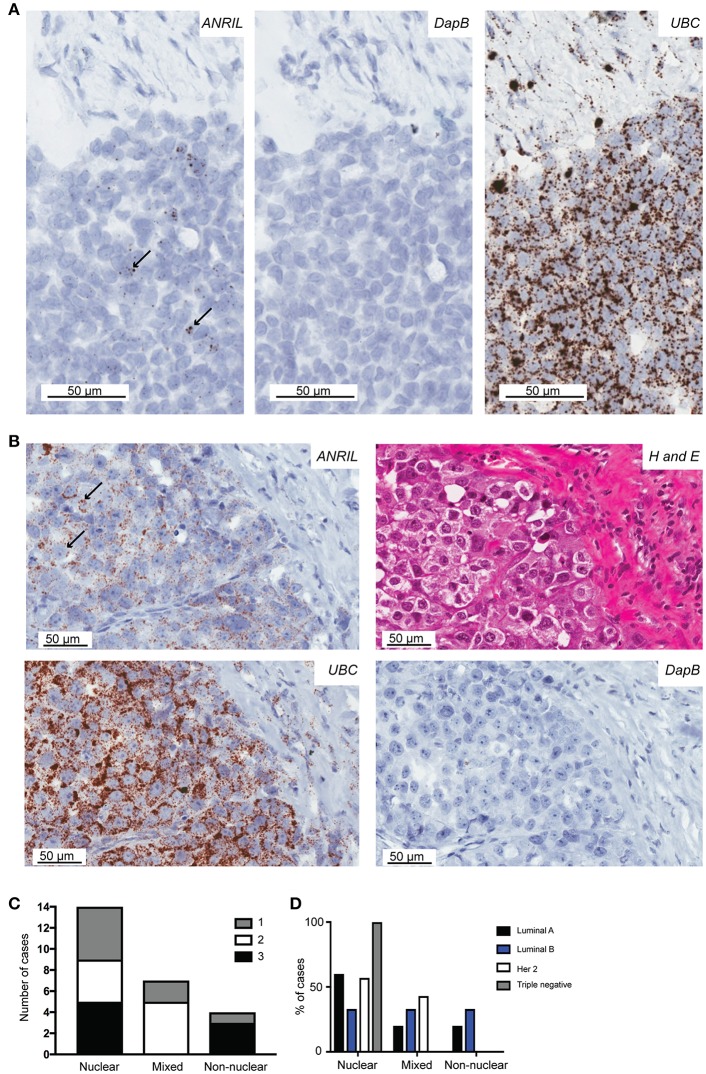
The localization of *ANRIL* in breast tumors using RNAscope *in situ* hybridization. Using RNAscope different subcellular localizations of *ANRIL* were revealed in breast tumors. **(A)** Left panel, Nuclear *ANRIL* (brown dots) in malignant cells; Middle panel, the negative control *DapB*; and Right panel, the positive control *UBC*. **(B)** Top left panel, Non-nuclear *ANRIL* (brown dots) in malignant cells; Top right, hematoxylin and eosin staining showing cellular morphology confirming presence of malignant cells; Bottom left, the positive control *UBC*; Bottom right, the negative control *DapB*. Magnification x400. **(C)** Summary of the *ANRIL* RNAscope assay results across all breast tumors analyze including the subcellular localization of the staining. The amount of staining was semi quantified using a score based on the number of positive dots and the number of positive cells (0 lowest-3 highest staining). **(D)** Summary of the *ANRIL* location results across all breast tumor subtypes.

To determine the cell type responsible for *ANRIL* expression in breast tumors, 25 cases with sufficient tissue available and those with *ANRIL* expressed >2.5-fold compared to the mean expression of the house keeping genes were selected and subjected to analyses using RNAscope. Cases included 5 Luminal A, 8 Luminal B, 4 triple negative, and 8 Her 2 positive tumors. Tissue sections were first assessed using a positive control probe for *UBC* to check for sufficient RNA quality and sequential sections stained for *ANRIL* and a negative control probe. All cases showed strong staining for *UBC* and variable amounts of *ANRIL* staining that was absent in the negative control. All cases showed the majority of *ANRIL* expression in malignant epithelial cells with no expression identified in normal mammary epithelium in the same section. In fifteen (60%) cases, scattered non-malignant cells were also positive for *ANRIL* and included endothelial cells (1–9 dots in positive cells and up to three cells positive focally) and other stromal cells (3–9 dots positive in individual cells). The RNAscope assay revealed different cellular localization for *ANRIL* (Figure 2) in malignant cells with staining in the nucleus (Figure 2A) and cytoplasm (Figure 2B). The majority of cases had *ANRIL* only in the nucleus (*n* = 14, 56% and Figure 2A top panel), 28% of cases had staining in the nucleus and outside of the nucleus (*n* = 7), and 16% of cases had *ANRIL* staining outside of the nucleus (*n* = 4, and Figure 2C). Although the number of tumors in individual tumor subtypes were limited. The majority of Luminal A and Her 2 and all triple negative breast tumors had *ANRIL* only in the nucleus. Luminal B tumors showed more of a mixed location for *ANRIL* with one third of tumors with *ANRIL* in the nucleus, one third with *ANRIL* outside the nucleus and one third with *ANRIL* in both the nucleus and outside the nucleus.

Overall these findings suggest *ANRIL* was largely present in malignant cells and its presence inside and outside of the nucleus suggests *ANRIL* is heterogeneously located amongst breast tumors.

### *ANRIL* Correlated With *POSTN* in Malignant Breast Cells

**Figure 3:**
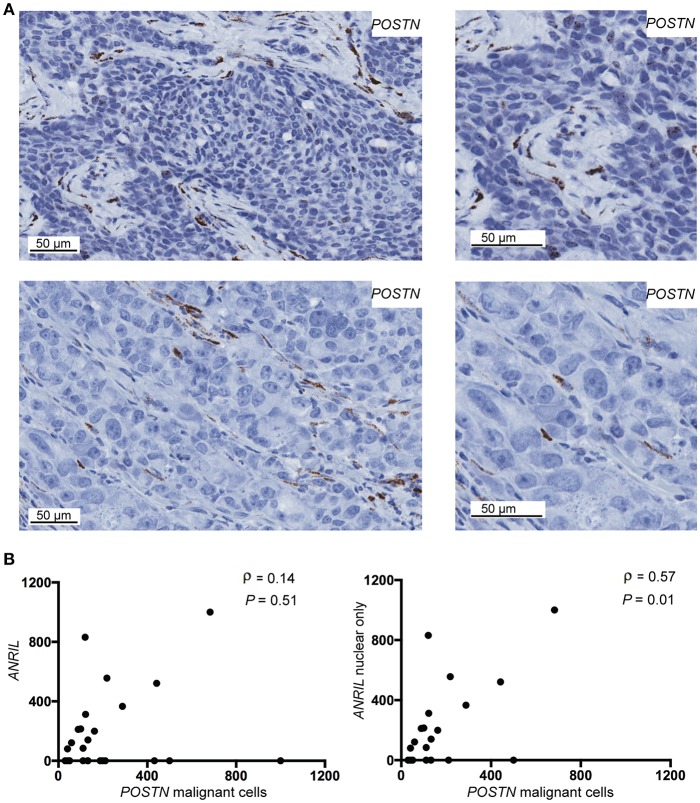
The correlation between *POSTN* and *ANRIL* in breast tumors using RNAscope *in situ* hybridization. **(A)** Using RNAscope *POSTN* was expressed in the malignant and stroma (top panel) or the stroma only (bottom panel) in breast tumors. Images at the same magnification but enlarged are shown to the right to better illustrate positively stained cells. **(B)** Scatter plot illustrating *ANRIL* and *POSTN* expression in malignant cells. Spearman's correlation coefficient (ρ) between *ANRIL* and *POSTN* revealed no association when all *ANRIL* expressing tumors were included in the analysis (left panel), but a significant correlation when cytoplasmic only expressing tumors were excluded (right panel).

To determine if malignant cells that expressed *ANRIL* also expressed *CCL2, IL6* or *POSTN*, the RNAscope analyses were extended to include each cytokine and the areas with *ANRIL* staining were correlated with the other markers. Only one tumor had *IL6* staining in malignant cells with *ANRIL* staining and no cases showed *CCL2* staining in cells with *ANRIL*. Areas with *ANRIL* positivity in malignant cells also showed *POSTN* staining (Figure 3A). An analysis including all *ANRIL* positive tumors found no correlation between *ANRIL* and *POSTN* (Figure 3B, left panel); however, a positive correlation was found when only tumors with nuclear staining were included (Figure 3B, right panel). These results suggest in breast tumors nuclear *ANRIL* is correlated with *POSTN* expression.

### *ANRIL* Correlated With *POSTN* in Breast Cell Lines

**Figure 4:**
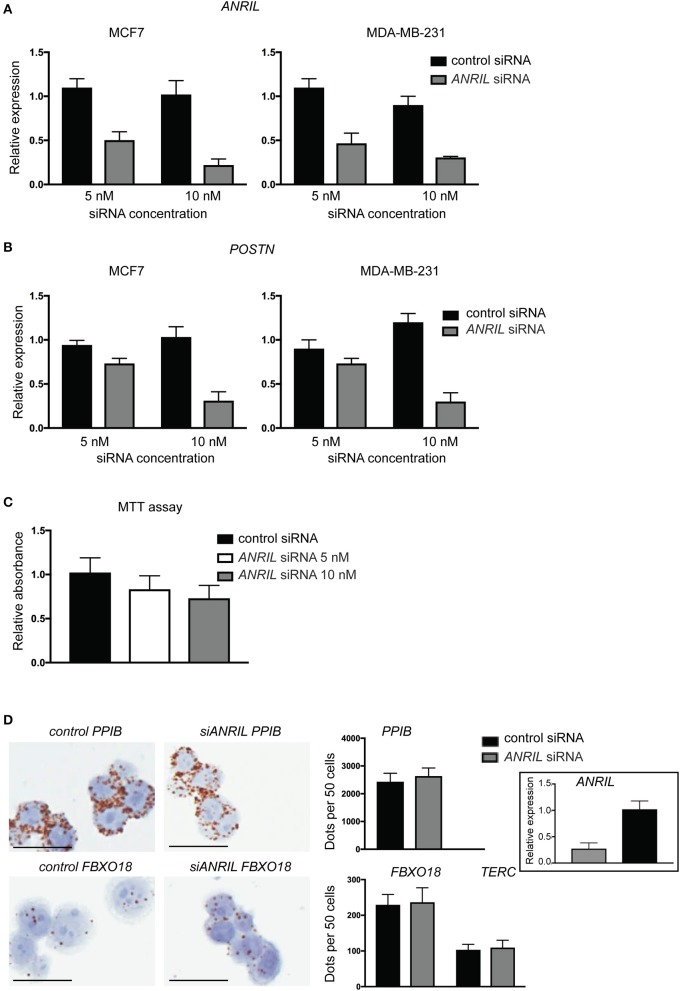
siRNA toward *ANRIL* in breast cancer cell lines is associated with reduced *POSTN* expression using RT-qPCR. **(A)** Breast cancer cell lines MCF7 and MDA-MB-231 were treated with siRNAs toward *ANRIL* or a negative control at two different concentrations. **(B)** Cells treated with siRNA toward *ANRIL* or the negative control were also measured for *POSTN*. All RT-qPCRs were normalized to *GAPDH and HPRT* and results expressed relative to expression found in untreated cells. **(C)** The MTT assay was performed on MCF7 cells to identify if *siANRIL* affected cell proliferation; no significance differences were found. **(D)** MCF7 cells were treated with siRNAs toward *ANRIL* or a negative control siRNA (control siRNA) at 10 nM, cells harvested, fixed, and processed into paraffin wax. Cell sections were subjected to RNAscope for genes not expected to be affected by *siANRIL* (*PPIB, FBXO18*, and *TERC*). No difference in positive staining (brown dots) was found in cells treated with the control siRNA compared to those treated with si*ANRIL*. Scale bar, 25 μm taken at 400x magnification. Data represented as mean ± SD for at least three replicates of each sample. Boxed insert siRNA for *PPIB* and *FBXO18* experiments.

**Figure 5:**
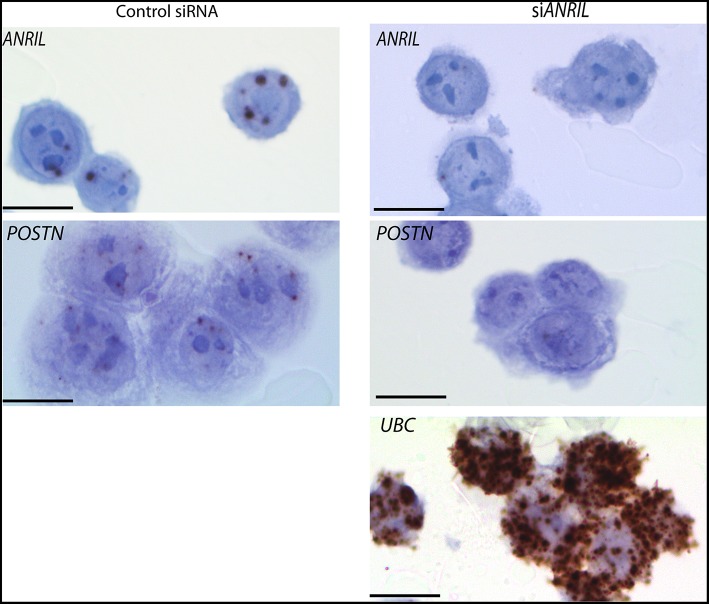
Reduced *POSTN* in MCF7 cells treated with siRNA toward *ANRIL* using RNAscope. The breast cancer cell line MCF7 was treated with siRNAs toward *ANRIL* or a negative control siRNA (control siRNA) at 10 nM, cells harvested, fixed, and processed into paraffin wax. Cell sections were subjected to RNAscope for *ANRIL, POSTN*, and *UBC*. Increased positive staining (brown dots) for *ANRIL* and *POSTN* was found in cells treated with the control siRNA. Scale bar, 20 μm taken at 1000x magnification under oil.

To test the correlation between *ANRIL* and *POSTN* found in breast tumors siRNA was used to knockdown *ANRIL* levels in two breast cancer cell lines and the effect on *POSTN* expression measured using RT-qPCR. Two cell lines were selected MDA-MB-231 and MCF7 cells that expressed *ANRIL* (26) In both cell lines *ANRIL* was knocked down relative to cells treated with the negative control siRNA (Figure 4A). Cells with reduced *ANRIL* also had reduced *POSTN* expression (Figure 4B). The MTT assay showed no significance difference between MCF7 cells treated with siANRIL and those treated with the control siRNA (Figure 4C). MCF7 cells treated with siRNA for *ANRIL* and the negative control siRNA were also investigated using RNAscope. There was no difference in expression of genes not expected to be affected by *ANRIL* (*PPIB, FBXO18* and *TERC)* in MCF7 cells treated with the si*ANRIL* compared to those treated with the control siRNA (Figure 4D). However, RNAscope for *ANRIL* and *POSTN* showed that although *ANRIL* and *POSTN* expression were low in cells treated with the negative control siRNA it was further reduced in cells treated with siRNA for *ANRIL* (Figure 5), consistent with the findings using RT-qPCR.

## Discussion

*ANRIL* is a LncRNA recently found to be elevated in a range of cancers including breast cancer. In this study, we applied an *in situ* analysis and identified *ANRIL* largely localized to malignant cells with heterogeneity in its subcellular location. The correlation between *POSTN* and *ANRIL* was found when only tumors with nuclear *ANRIL* were included, suggesting the subcellular localization of *ANRIL* will be important in determining the potential effects of *ANRIL* toward cancer progression.

Further support toward *ANRIL* being a potential biomarker for breast cancer was evident with elevated *ANRIL* in the tumor compared to the normal adjacent tissue. Elevated *ANRIL* is most commonly found in triple negative breast tumors (12, 14, 16). The finding of elevated *ANRIL* in the tumor when compared to normal adjacent tissue from the same donor irrespective of the subtype suggests *ANRIL* may be more universally increased in breast cancer, but the highest levels occur in triple negative tumors. Finding *ANRIL* predominately in malignant cells provides further support toward *ANRIL* being a biomarker in breast cancer.

*ANRIL* may contribute to the tumor microenvironment by supplying a protein that remodels the extracellular matrix, promoting metastasis (27). A correlation between *ANRIL* and *POSTN* has been found in an investigation of genetic risk for cardiovascular disease (10). Periostin induces many other cancer promoting abilities when administered to various cell lines (27). The finding of nuclear but not cytoplasmic *ANRIL* associated with *POSTN* is consistent with different functions of *ANRIL*. One of the best-known mechanisms for *ANRIL* is gene silencing via epigenetic regulation involving recruiting and binding to the components of polycomb group of transcriptional repressor proteins (PcG), namely, PRC1 and PRC2 (14). Using chromatin fractionation, although not exclusively for *ANRIL* Ray et al. (28) found a number of lncRNAs involved in PcG biology to be primarily localized to the nucleus (28), and in a second study nuclear extracts of prostate cancer cells showed *ANRIL* association with CBX7 protein a member of the PRC1 complex (5). The tumor microenvironment may increase *ANRIL*. In fibroblast and osteosarcoma cell lines, *ANRIL* levels rise in response to a variety of DNA damaging agents via an ATM-induced E2F1 transcription-mediated pathway (29) and *ANRIL* contributes to pro-inflammatory cytokine expression by binding to YingYang-1 (YY1) in response to NFκB signaling (8). *POSTN* is regulated by YY1, so it is possible that periostin may be an additional cytokine to IL6/8 regulated by the YY1/*ANRIL* complex (30). Considering periostin is commonly produced by stromal cells the relevance of the contribution from malignant cells is unclear. In breast cancer *POSTN* was elevated in malignant cells and associated with poorer patient progression free and overall survival (31). In a second study *POSTN* was associated with a poorer prognosis in triple negative tumors (32). However, in other studies *POSTN* in stromal cells was thought to be important in breast cancer progression (32, 33).

The subcellular localization of *ANRIL* between breast tumors was heterogeneous. In an extensive survey of 61 LncRNA that included *ANRIL* in three cell lines revealed that individual LcRNAs showed cell-to-cell variability further extending the benefits of visualization LcRNAs directly (34). In cancer cell lines *ANRIL* was located in the nucleus with one or two large foci (34, 35) or in both the nucleus or cytoplasmic fraction (36). In non-transformed cells human umbilical vein endothelial cells (HUVEC) and normal lung tissue *ANRIL* was present in the nucleus and cytoplasm including perinuclear cytoplasmic staining (37, 38). Different isoforms of *ANRIL* exist, linear (exons 1–3, 15–20) or circular (exons 4–14) forms (39). In melanoma cell lines, linear isoforms of *ANRIL* were specifically found to be enriched in the nucleus and circular isoforms enriched in the cytoplasmic (36). The *ANRIL* probe designed by RNAscope spans from base pairs 9-1252 (exons 1–6) spanning exons in both the linear and cytoplasmic isoforms, thus the different subcellular localization found in breast tumors may reflect different isoforms being present.

## Conclusion

This study highlights that *ANRIL* exists in different subcellular locations in breast tumors, which may affect its functionality, for example whether nuclear *ANRIL* is associated with increased *POSTN* expression in malignant epithelial cells.

## Data Availability

The raw data supporting the conclusions of this manuscript will be made available by the authors, without undue reservation, to any qualified researcher.

## Ethics Statement

Ethics Committee Approval LRS/10/09/035.

## Author Contributions

PM-M performed the RNAscope and qPCR analyzes, analyzed and interpreted the data, and co-wrote the manuscript. HC designed some of the analyses, interpreted the data, and edited the manuscript. NH designed the study, analyzed and interpreted the data, edited the manuscript, and obtained the ethical approval. TS conceived the study, analyzed and interpreted the data, obtained the funding, and co-wrote the manuscript.

### Conflict of Interest Statement

The authors declare that the research was conducted in the absence of any commercial or financial relationships that could be construed as a potential conflict of interest.

## References

[1] Sethi N Kang Y. Unravelling the complexity of metastasis - molecular understanding and targeted therapies. Nat Rev Cancer. (2011) 11:735–48. 10.1038/nrc3125 21941285 PMC10961136

[2] Schmitt AM Chang HY. Long noncoding RNAs in cancer pathways. Cancer Cell. (2016) 29:452–63. 10.1016/j.ccell.2016.03.010 27070700 PMC4831138

[3] Yang G Lu X Yuan L. LncRNA: a link between RNA and cancer. Biochim Biophys Acta. (2014) 1839:1097–109. 10.1016/j.bbagrm.2014.08.012 25159663

[4] Aguilo F Cecilia SD Walsh MJ. Long non-coding RNA ANRIL and polycomb in human cancers and cardiovascular disease. Curr Top Microbiol Immunol. (2016) 394:29–39. 10.1007/82_2015_455 26220772 PMC4732918

[5] Yap KL Li S Munoz-Cabello AM Raguz S Zeng L Mujtaba S. Molecular interplay of the noncoding RNA ANRIL and methylated histone H3 lysine 27 by polycomb CBX7 in transcriptional silencing of INK4a. Mol Cell. (2010) 38:662–74. 10.1016/j.molcel.2010.03.021 20541999 PMC2886305

[6] Pasmant E Laurendeau I Heron D Vidaud M Vidaud D Bieche I. Characterization of a germ-line deletion, including the entire INK4/ARF locus, in a melanoma-neural system tumor family: identification of ANRIL, an antisense noncoding RNA whose expression coclusters with ARF. Cancer Res. (2007) 67:3963–9. 10.1158/0008-5472.CAN-06-2004 17440112

[7] Qiao C Yang L Wan J Liu X Pang C You W. Long noncoding RNA ANRIL contributes to the development of ulcerative colitis by miR-323b-5p/TLR4/MyD88/NF-kappaB pathway. Biochem Biophys Res Commun. (2018) 508:217–24. 10.1016/j.bbrc.2018.11.100 30477744

[8] Zhou X Han X Wittfeldt A Sun J Liu C Wang X. Long non-coding RNA ANRIL regulates inflammatory responses as a novel component of NF-κB pathway. RNA Biol. (2016) 13:98–108. 10.1080/15476286.2015.1122164 26618242 PMC4829310

[9] Zhang B Wang D Ji TF Shi L Yu JL. Overexpression of lncRNA ANRIL up-regulates VEGF expression and promotes angiogenesis of diabetes mellitus combined with cerebral infarction by activating NF-kappaB signaling pathway in a rat model. Oncotarget. (2017) 8:17347–59. 10.18632/oncotarget.14468 28060742 PMC5370045

[10] Pilbrow AP Folkersen L Pearson JF Brown CM McNoe L Wang NM. The chromosome 9p21.3 coronary heart disease risk allele is associated with altered gene expression in normal heart and vascular tissues. PLoS ONE. (2012) 7:e39574. 10.1371/journal.pone.0039574 22768093 PMC3387158

[11] Turnbull C Ahmed S Morrison J Pernet D Renwick A Maranian M. Genome-wide association study identifies five new breast cancer susceptibility loci. Nat Genet. (2010) 42:504–7. 10.1038/ng.586 20453838 PMC3632836

[12] Iranpour M Soudyab M Geranpayeh L Mirfakhraie R Azargashb E Movafagh A. Expression analysis of four long noncoding RNAs in breast cancer. Tumour Biol. (2015) 37:2933–40. 10.1007/s13277-015-4135-2 26409453

[13] Royds JA Pilbrow AP Ahn A Morrin HR Frampton C Russell IA. The rs11515 polymorphism is more frequent and associated with aggressive breast tumors with increased ANRIL and decreased p16 (INK4a) expression. Front Oncol. (2015) 5:306. 10.3389/fonc.2015.00306 26835415 PMC4720739

[14] Meseure D Vacher S Drak Alsibai K Nicolas A Chemlali W Caly M. Expression of ANRIL - polycomb complexes - CDKN2A/B/ARF genes in breast tumors: identification of a two-gene (EZH2/CBX7) signature with independent prognostic value. Mol Cancer Res. (2016) 14:623–33. 10.1158/1541-7786.MCR-15-0418 27102007

[15] Liu M L.-Xing Q Liu JY. A three-long noncoding RNA signature as a diagnostic biomarker for differentiating between triple-negative and non-triple-negative breast cancers. Medicine. (2017) 96:e6222. 10.1097/MD.0000000000006222 28248879 PMC5340452

[16] Xu ST Xu JH Zheng ZR Zhao QQ Zeng XS Cheng SX. Long non-coding RNA ANRIL promotes carcinogenesis via sponging miR-199a in triple-negative breast cancer. Biomed Pharmacother. (2017) 96:14–21. 10.1016/j.biopha.2017.09.107 28961506

[17] Zhang Z Feng L Liu P Duan W. ANRIL promotes chemoresistance via disturbing expression of ABCC1 by regulating the expression of Let-7a in colorectal cancer. Biosci Rep. (2018) 38:BSR20180620. 10.1042/BSR20180620 30279206 PMC6246772

[18] Zhang EB Kong R Yin DD You LH Sun M Han L. Long noncoding RNA ANRIL indicates a poor prognosis of gastric cancer and promotes tumor growth by epigenetically silencing of miR-99a/miR-449a. Oncotarget. (2014) 5:2276–92. 10.18632/oncotarget.1902 24810364 PMC4039162

[19] Paul Y Thomas S Patil V Kumar N Mondal B Hegde AS. Genetic landscape of long noncoding RNA (lncRNAs) in glioblastoma: identification of complex lncRNA regulatory networks and clinically relevant lncRNAs in glioblastoma. Oncotarget. (2018) 9:29548–64. 10.18632/oncotarget.25434 30038703 PMC6049862

[20] Tsuyada A Chow A Wu J Somlo G Chu P Loera S. CCL2 mediates cross-talk between cancer cells and stromal fibroblasts that regulates breast cancer stem cells. Cancer Res. (2012) 72:2768–79. 10.1158/0008-5472.CAN-11-3567 22472119 PMC3367125

[21] Kumari N Dwarakanath BS Das A Bhatt AN. Role of interleukin-6 in cancer progression and therapeutic resistance. Tumour Biol. (2016) 37:11553–72. 10.1007/s13277-016-5098-7 27260630

[22] Gonzalez-Gonzalez L Alonso J. Periostin: a matricellular protein with multiple functions in cancer development and progression. Front Oncol. (2018) 8:225. 10.3389/fonc.2018.00225 29946533 PMC6005831

[23] Tang P Tse GM. Immunohistochemical surrogates for molecular classification of breast carcinoma: a 2015 update. Arch Pathol Lab Med. (2016) 140:806–14. 10.5858/arpa.2015-0133-RA 27472239

[24] Li J Smyth P Cahill S Denning K Flavin R Aherne S Improved RNA quality and TaqMan(®)Pre-amplification method (PreAmp) to enhance expression analysis from formalin fixed paraffin embedded (FFPE) materials. BMC Biotechnol. (2008) 8:10 10.1186/1472-6750-8-10 18254955 PMC2259333

[25] Schmittgen TD Livak KJ Analyzing real-time PCR data by the comparative CT method. Nat Protocols. (2008) 3:1101–8. 10.1038/nprot.2008.73 18546601

[26] Lee J Jung JH Chae YS Park HY Kim WW Lee SJ. Long noncoding RNA snaR regulates proliferation, migration and invasion of triple-negative breast cancer cells. Anticancer Res. (2016) 36:6289–95. 10.21873/anticanres.11224 27919948

[27] González-González L Alonso J. Periostin: a matricellular protein with multiple functions in cancer development and progression. Front. Oncol. (2018) 8:225. 29946533 10.3389/fonc.2018.00225 PMC6005831

[28] Ray MK Wiskow O King MJ Ismail N Ergun A Wang Y. CAT7 and cat7l Long Non-coding RNAs tune polycomb repressive complex 1 function during human and zebrafish development. J Biol Chem. (2016) 291:19558–72. 10.1074/jbc.M116.730853 27405765 PMC5016691

[29] Wan G Mathur R Hu X Liu Y Zhang X Peng G. Long non-coding RNA ANRIL (CDKN2B-AS) is induced by the ATM-E2F1 signaling pathway. Cell Signal. (2013) 25:1086–95. 10.1016/j.cellsig.2013.02.006 23416462 PMC3675781

[30] Romeo F Falbo L Di Sanzo M Misaggi R Faniello MC Barni T. Negative transcriptional regulation of the human periostin gene by YingYang-1 transcription factor. Gene. (2011) 487:129–34. 10.1016/j.gene.2011.07.025 21839814

[31] Kim GE Lee JS Park MH Yoon JH. Epithelial periostin expression is correlated with poor survival in patients with invasive breast carcinoma. PLoS ONE. (2017) 12:e0187635. 10.1371/journal.pone.0187635 29161296 PMC5697858

[32] Lambert AW Wong CK Ozturk S Papageorgis P Raghunathan R Alekseyev Y. Tumor cell-derived periostin regulates cytokines that maintain breast cancer stem cells. Mol Cancer Res. (2016) 14:103–13. 10.1158/1541-7786.MCR-15-0079 26507575 PMC4715959

[33] Ratajczak-Wielgomas K Grzegrzolka J Piotrowska A Matkowski R Wojnar A Rys J. Expression of periostin in breast cancer cells. Int J Oncol. (2017) 51:1300–10. 10.3892/ijo.2017.4109 28902360

[34] Cabili MN Dunagin MC McClanahan PD Biaesch A Padovan-Merhar O Regev A. Localization and abundance analysis of human lncRNAs at single-cell and single-molecule resolution. Genome Biol. (2015) 16:20. 10.1186/s13059-015-0586-4 25630241 PMC4369099

[35] Shinjo K Yamashita Y Yamamoto E Akatsuka S Uno N Kamiya A. Expression of chromobox homolog 7 (CBX7) is associated with poor prognosis in ovarian clear cell adenocarcinoma via TRAIL-induced apoptotic pathway regulation. Int J Cancer. (2014) 135:308–18. 10.1002/ijc.28692 24375438

[36] Sarkar D Oghabian A Bodiyabadu PK Joseph WR Leung EY Finlay GJ. Multiple isoforms of ANRIL in melanoma cells: structural complexity suggests variations in processing. Int J Mol Sci. (2017) 18:1378. 10.3390/ijms18071378 28653984 PMC5535871

[37] Thomas AA Feng B Chakrabarti S. ANRIL: a regulator of VEGF in diabetic retinopathy. Invest Ophthalmol Visual Sci. (2017) 58:470–80. 10.1167/iovs.16-20569 28122089

[38] Kang YH Kim D Jin EJ. Down-regulation of phospholipase D stimulates death of lung cancer cells involving up-regulation of the long ncRNA ANRIL. Anticancer Res. (2015) 35:2795–803. 25964559

[39] Burd CE Jeck WR Liu Y Sanoff HK Wang Z Sharpless NE. Expression of linear and novel circular forms of an INK4/ARF-associated non-coding RNA correlates with atherosclerosis risk. PLoS Genet. (2010) 6:e1001233. 10.1371/journal.pgen.1001233 21151960 PMC2996334

